# Long-Term Retrospective Analysis of Mackerel Spawning in the North Sea: A New Time Series and Modeling Approach to CPR Data

**DOI:** 10.1371/journal.pone.0038758

**Published:** 2012-06-21

**Authors:** Teunis Jansen, Kasper Kristensen, Mark Payne, Martin Edwards, Corinna Schrum, Sophie Pitois

**Affiliations:** 1 Technical University of Denmark (DTU) AQUA, National Institute of Aquatic Resources Charlottenlund, Charlottenlund, Denmark; 2 Sir Alister Hardy Foundation for Ocean Science (SAHFOS), Plymouth, United Kingdom; 3 Geophysical Institute, University of Bergen, Bergen, Norway; 4 Centre of Environment, Fisheries & Aquaculture Science (CEFAS), Suffolk, United Kingdom; University of Vigo, Spain

## Abstract

We present a unique view of mackerel (*Scomber scombrus*) in the North Sea based on a new time series of larvae caught by the Continuous Plankton Recorder (CPR) survey from 1948-2005, covering the period both before and after the collapse of the North Sea stock. Hydrographic backtrack modelling suggested that the effect of advection is very limited between spawning and larvae capture in the CPR survey. Using a statistical technique not previously applied to CPR data, we then generated a larval index that accounts for both catchability as well as spatial and temporal autocorrelation. The resulting time series documents the significant decrease of spawning from before 1970 to recent depleted levels.

Spatial distributions of the larvae, and thus the spawning area, showed a shift from early to recent decades, suggesting that the central North Sea is no longer as important as the areas further west and south. These results provide a consistent and unique perspective on the dynamics of mackerel in this region and can potentially resolve many of the unresolved questions about this stock.

## Introduction

Mackerel (*Scomber scombrus*) is one of the most abundant and widely distributed fish species in the North East Atlantic [1]. Mackerel plays an important ecological role by feeding on zooplankton and on the pelagic larval and juvenile stages of a number of commercially important fish stocks [2]. Mackerel is furthermore caught by a large pelagic fishery with annual landings between 500 and 1000 thousand tonnes [1]. Large changes in mackerel abundance and distribution have therefore significant effects on ecosystems as well as economies. The ecological impact through altered predation pressures on secondary production and fish recruits are likely large, but currently not assessed [2]. More easily observed are the political and economic consequences [3], [4].

**Figure 1:**
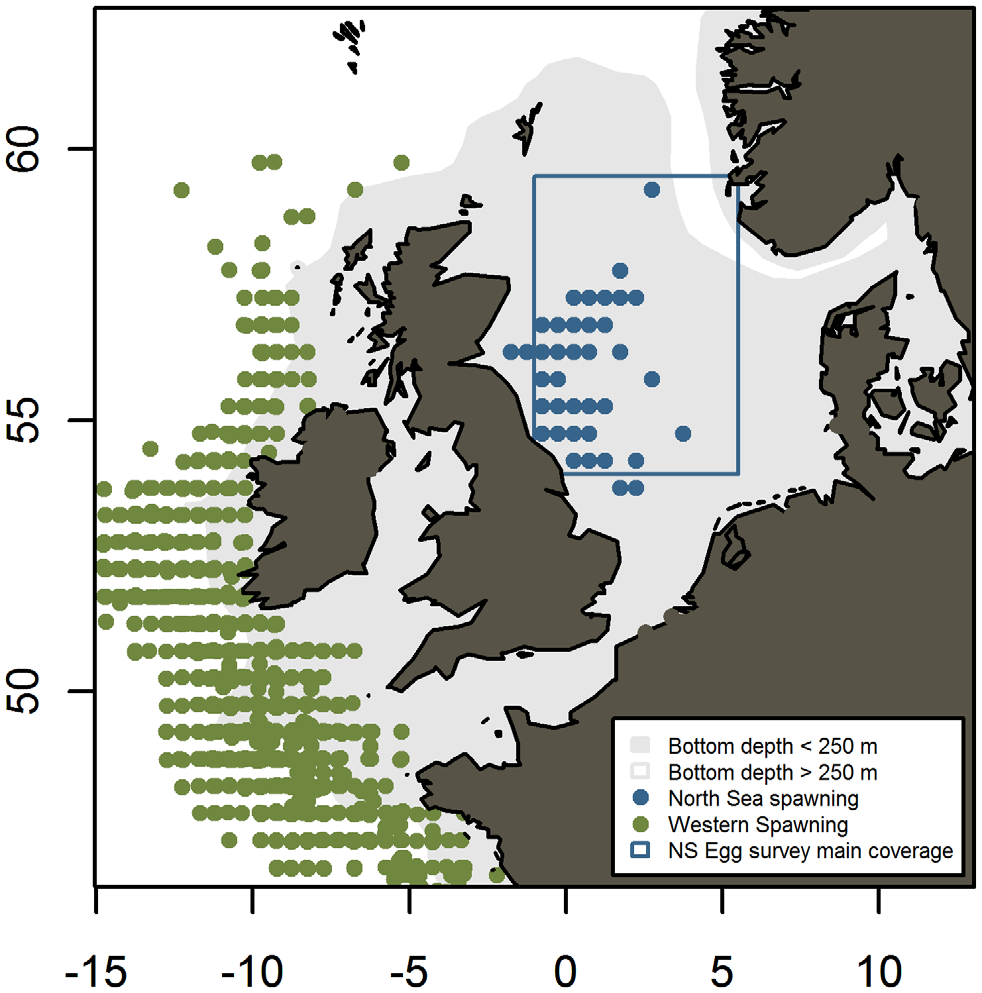
Mackerel populations and distribution around the north-western European shelf. Continental shelf marked in grey (bottom depth <250 m). North Sea and western mackerel spawning areas indicated by dots. Each dot marks an observation of 50+ eggs m^−2^ day^−1^. Data from international mackerel egg surveys (Blue = North Sea 2002–2011 [7], [34], [35], Green = Western areas 1977–2007 (ICES WGMEGS)). Blue rectangle marks the approximate main coverage of the international mackerel egg survey in the latter years [7], [34], [35].

Radical changes in abundance and distribution have been observed throughout the north-east Atlantic during the last century of developing mackerel science and fisheries [1] especially in the North Sea. The North Sea mackerel is considered to be a distinct stock that, unlike the western mackerel stock spawns inside the North Sea (Figure 1). The North Sea spawning stock was large and lightly fished up to the late 1960s, where the development of modern sonars, power blocks and single-vessel purse seining led to a ten-fold increase in mackerel landings [5]. This fishery was unsustainable and resulted in a collapse of the stock in the 1970s. Despite subsequent regulations of the fishery designed specifically to protect this stock, it never rebuilt to its former level. In the last decade the spawning stock biomass has been 150-230 kt [1], compared to over 2 500 kt in the beginning of the 1960s [6], [7]. It is currently unknown why the North Sea stock has not rebuilt to former levels.

Unfortunately, documentation of the historic development is based on fragmented information sources that do not consistently cover the whole period from before to after the collapse. This is a hindrance for addressing key questions about the lack of stock rebuilding and the consequences of these changes in distribution and abundance. An internally-consistent time-series with broad temporal span would therefore greatly aid the understanding of the development of this stock.

One such potential time series stems from the Continuous Plankton Recorder (CPR) survey in the North Sea. The CPR is a self-contained automatic plankton recorder that collects plankton continuously while being pulled by route-vessels of opportunity e.g. ferries. The monthly deployment on a variety of routes through 8 decades have resulted in a unique time series that have been a cornerstone in studies of long term-trends in the North Sea for a range of lower trophic plankton organisms [8].

Recently the analysis of fish larvae in the CPR samples has been completed up to 2005. This offer a unique opportunity to investigate long term changes in abundance and distribution of mackerel larvae.

We present here the new time series of mackerel abundance in the North Sea based upon larvae caught by the CPR from 1948 to 2005, spanning both the period prior to the development of the intensive fishing in the late 1960s and modern times. We verify the spatial origin of the larvae through use of a hydrographic backtracking model for all sampled larvae. Using a technique not previously applied to CPR data, we then construct a larvae index considering catchability as well as spatial and temporal autocorrelation. Considering the larvae abundance as a proxy for number of spawned eggs and spawner biomass, we compare it with existing egg survey data and fisheries-based assessments with a focus on the decline around the 1970’s. We review the possible applications of this time series, including supplementing or improving the mackerel stock assessment and the international mackerel egg survey with data from the CPR survey. Finally, we provide recommendations regarding calculation procedures for CPR data.

## Materials and Methods

### Mackerel Larvae Data

Mackerel larvae from Continuous Plankton Recorder (CPR) surveys in 1948 to 2005 in the region 51–61°N and 3.5°W–9.5°E were obtained from the SAHFOS database. The details of the CPR survey are described elsewhere [9], [10]. Briefly, the CPR are towed by ships of opportunity at speeds in the range of 10–15 knots and at an approximate depth of 7 m [9], [11]. Water enters the recorder through an aperture of 1.62 cm^2^, and is filtered through a continuously moving band of silk with an average mesh size of 270 µm. The captured plankton is fixed in formalin. The silk band is divided into samples representing 10 miles of tow for analysis, equivalent to approximately 3 m^3^ of filtered seawater. Methods of counting and data processing are described by [9], [10].

### Thermocline Data

Thermocline depth data for the period 1948–2005 were processed from a long-term ECOSMO model run [12], [13]. The model is a coupled physical-biological 3-d deterministic model. It simulates the time varying hydrodynamic and lower trophic level conditions in the region North Sea and Baltic Sea as a function of atmospheric, oceanic and terrestrial time varying boundary conditions. The thermocline data are provided on the spherical model grid (0.1° lat × 1/6° lon) as monthly averages. Similar data from an earlier model simulation [14] are available via the ICES WGOOFE website (www.wgoofe.org) or directly from the University of Bergen (ftp://ftp.gfi.uib.no/pub/gfi/corinna/WGOOFE/only_physics_run_1958 2004/ASCII/monthly/NSea/).

### Effect of Larval Drift

The positions of mackerel larvae captured by the CPR survey do not necessarily correspond to the actual location where spawning took place. Icthyoplankton can, in some regions of the North Sea, be rapidly advected away from their spawning location: the magnitude and direction of this drift can vary appreciably between years (e.g [15], [16]). As a first step in the analysis of the larval dataset, we attempted to estimate the magnitude of this advection, and thereby check for a potential bias introduced by drift processes.

As the basis for these calculations we applied an established hydrographic backtracking technique [17], [18]. The backtracking calculation was performed using the IBMlib library [17], forced with hourly physical fields (currents, temperature and turbulence) derived from the NORWECOM model [19], [20]. These fields were available from 1970 to 2005. Larval observations outside this period were not modelled. For each location (in time and space) where Mackerel larvae where observed in the CPR survey, 100 particles representing mackerel “larvae” were released in the model, uniformly distributed throughout the water column. Time in the model was then run backwards to determine a range of possible trajectories along which the larvae could have originated. No active-behaviour was applied to the particles – the “larvae” were mixed throughout the water column following the modelled turbulence as passive tracers. No explicit attempt was made to account for ontogenetic changes during this time (e.g. changes in egg buoyancy, hatching of eggs, changes from endogenous to exogenous feeding of larvae).

The duration of the backwards-advection scheme was based upon an estimate of time-since-spawning. Mackerel larvae in the CPR survey have a mean length of 4.8 mm (s.d. 2.0 mm) [21]. Under good temperature and food conditions, mackerel larvae grow from a typical hatch size of 3 mm to 4.8 mm in approximately 2.4 days [22]. Mackerel eggs are pelagic and therefore drift of the eggs also needs to be accounted for: typically 50% of mackerel eggs have hatched after 6.7 days at 11°C [23]. We therefore estimate that, on average, approximately 10 days have passed since the larvae captured by the CPR were spawned.

The simulated mackerel particles were therefore advected backwards in time for 10 days. At the completion of this period the geographical distance between the site of capture and the end point was calculated was calculated for each particle and the median of the distance distribution calculated. The process was then repeated for all larval observations in the CPR and the distribution of advection-distances across all observations generated. This distribution was then used to assess the magnitude and importance of advection processes in shaping the distribution of larvae.

### Mackerel Larvae Model

#### The log gaussian cox model

The distribution of larvae captured in the CPR survey were analysed using the so-called “log-gaussian cox process” (LGCP) model [24]. This model assumes that observed larvae counts are Poisson distributed with a multivariate log-normal mean and a spatio-temporal correlation structure. Denote by *i* the id of the CPR sample and let N*_i_* be the number of larvae caught in the sample. The model then states that given an unobserved/latent log-intensity in *i* we have.
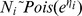



Note that exponentiating the random variable *η_i_* introduces overdispersion in the distribution [24] and that the latent vector *η* is assumed to be multivariate Gaussian.
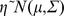
with a mean vector *µ* and covariance matrix Σ. The *µ* parameter describes the systematic effects while the covariance matrix models the random effects. Each sample unit *i* is associated with a set of covariates; position (cells of 0.3° latitude×0.6° longitude), year, day of year, thermocline depth and hour of day.

#### The random versus systematic effects

The spatio-temporal distribution of larvae is not completely random: aggregation in both space (“patches”) and time can be expected. Also, some degree of continuity from day to day and from year to year would be expected because the abundance of larvae are expected to be related to the stock size of the mackerel and mackerel lives and spawns for multiple years. We therefore consider the distribution of larvae as a so-called space-time separable random field with exponential correlation structure
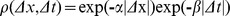
to define the covariance matrix Σ by



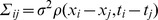



In words this means that if we consider two samples *i* and *j* then the correlation between the two log-abundances depends in an exponentially-decaying manner on the spatial distance between the samples (Δ*x*) and the temporal distance between the samples (Δ*t*), where larger distances have smaller correlations. The decay of the correlation in space and time is described by the model parameters *α* and *β*. The variance parameter *σ^2^* describes the variations from the high abundance to low abundance areas.

In reality, even if a sample is taken in an area with high abundance, it is not guaranteed that the catch will be high. This is because individual samples from the sea generally show a high level of small scale variability. We can account for this by adding a further level of variance at the sample level. This local noise effect is also referred to as the “nugget effect” *η_nugget_*_(*i*)_ [24].

It is assumed that spawning and hence larvae abundance follows a fixed seasonal pattern within the year, modelled here as a gaussian. However, the yearly level is considered as a random effect:
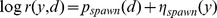
where *r*(*y, d*) is the number of larvae on day number *d* in year *y*. The seasonal log-abundance pattern is the 2^nd^ order polynomial *p_spawn_(d)*. Note that a 2nd order polynomial is the logarithm of a gaussian density. The yearly log-level of the abundance is the random variable *η(y)* which is assumed to be normal distributed with mean zero and variance *σ^2^*. A year to year correlation of this process is incorporated as exponentially decaying with the distance between years.

Due to the fact that the CPR operates horizontally in a fixed depth of approximately 7 m [9], [11] the catchability (the relationship between the number of larvae present in the water column and the number of larvae caught) of the recorder can be expected to be sensitive to changes in vertical distribution of the larvae. Small mackerel larvae, such as those caught by CPR, have been observed to stay above the thermocline where they migrate towards the surface at night [25], [26]. However, the water immediately behind a large, fast-moving vessel is likely to be mixed and homogenized well below the CPR towing depth [9]. To test and account for any systematic effects from changes in vertical distribution, we included diurnal migration (*µ*(*h*)) and thermocline depth (*p_thc_*(*thcl_i_*)) in the model. Non-significant (*p*>0.05) parameters were removed from the model. Furthermore, active avoidance of the sampling gear can also potentially affect catchability. This is more pronounced for larger larvae [26], but since the larvae caught by the CPR are small, we assumed that this effect was negligible.

#### Model summary

The log-intensity of individuals for sample number *i* taken at position *x*, year *y*, day number *d*, hour *h* is
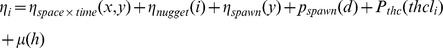
where

*η_space x time_* (*x, y*) is a mean zero gaussian stochastic process with covariance matrix

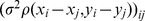

_._

*- η_nugget_*(*i*) is mean zero gaussian noise with variance 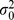.

*- η_spawn_*(*y*) is a mean zero stochastic process with covariance matrix (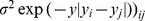
_._

*- p_spawn_*(*d*) is a second order polynomial 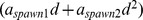 in the day number, d.

*- p_thc_*(*thcl_i_*) is a second order polynomial 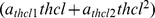 where *thcl_i_* is the thermocline depths at sample *i*.

*µ*(*h*) is a parameter vector with one level for each hour of the day.

#### Fitting the model

The model was fitted as in [24] by the maximum likelihood method using the Laplace approximation. It is an important feature of the approach that it can deal consistently with missing data: latent variables (no direct observation) are integrated out of the likelihood function. Furthermore a “best guess” of any latent variable can be reconstructed based on the likelihood function. More precisely we used the conditional expectation of the variable given the data. This estimator has the property of being unbiased and having smaller variance than any other unbiased estimator [24].

The fitted model was used to predict the larvae concentration at any point in the North Sea, through each day in the period 1948–2005. From this dataset we produced yearly distribution maps and a time series of yearly indices of larvae abundance, by calculating the posterior mean of the spatially integrated intensity for each year. The hypothesis of a change in abundance from before 1970 to after 1990 was tested by a likelihood-ratio hypothesis test.

The model was run in R v.2.13.1 with the package “lgc”. This package was developed in R and C and is available on request to kaskr@aqua.dtu.dk.

The annual larvae abundance index was compared to estimates of egg numbers and spawning stock size taken from the ICES WGWIDE reports and following publications [6], [27]–[29].

## Results

**Figure 2:**
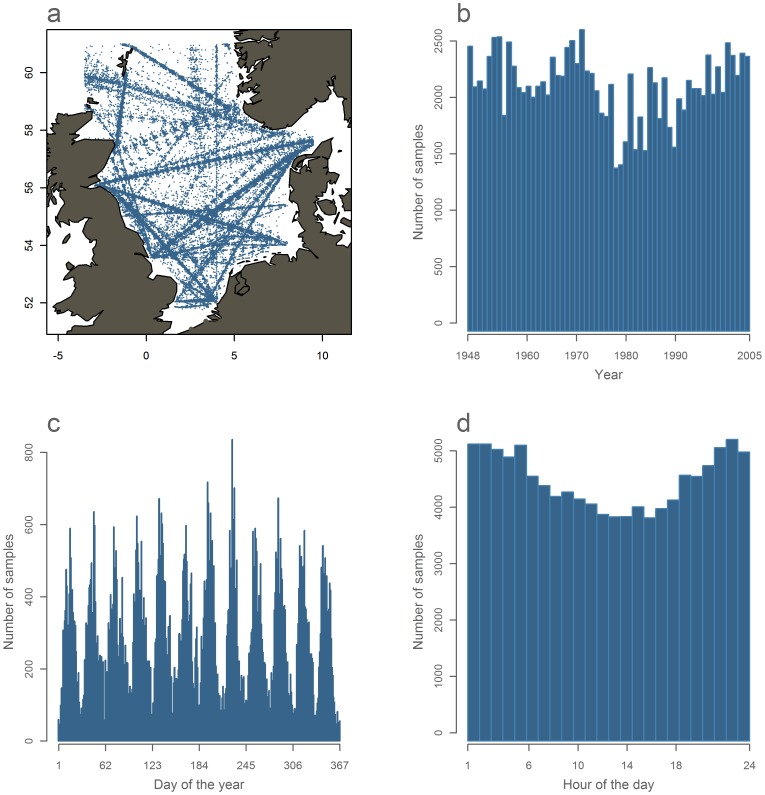
Continuous plankton recorder samples from 1948–2005 in the studied area. a) map of samples locations. b) number of samples by year. c) number of samples by day number of the year. d) number of samples by the hour of the day.

The CPR dataset consisted of 129,764 samples with 4,642 larvae observations. The samples are broadly distributed throughout the North Sea region (Figure 2a) and fairly equally distributed over the years (Figure 2b), within each year (Figure 2c) and day (Figure 2d). However, the sampling effort was poor in the central North Sea in the last decade of the time series (Figure S1).

**Figure 3:**
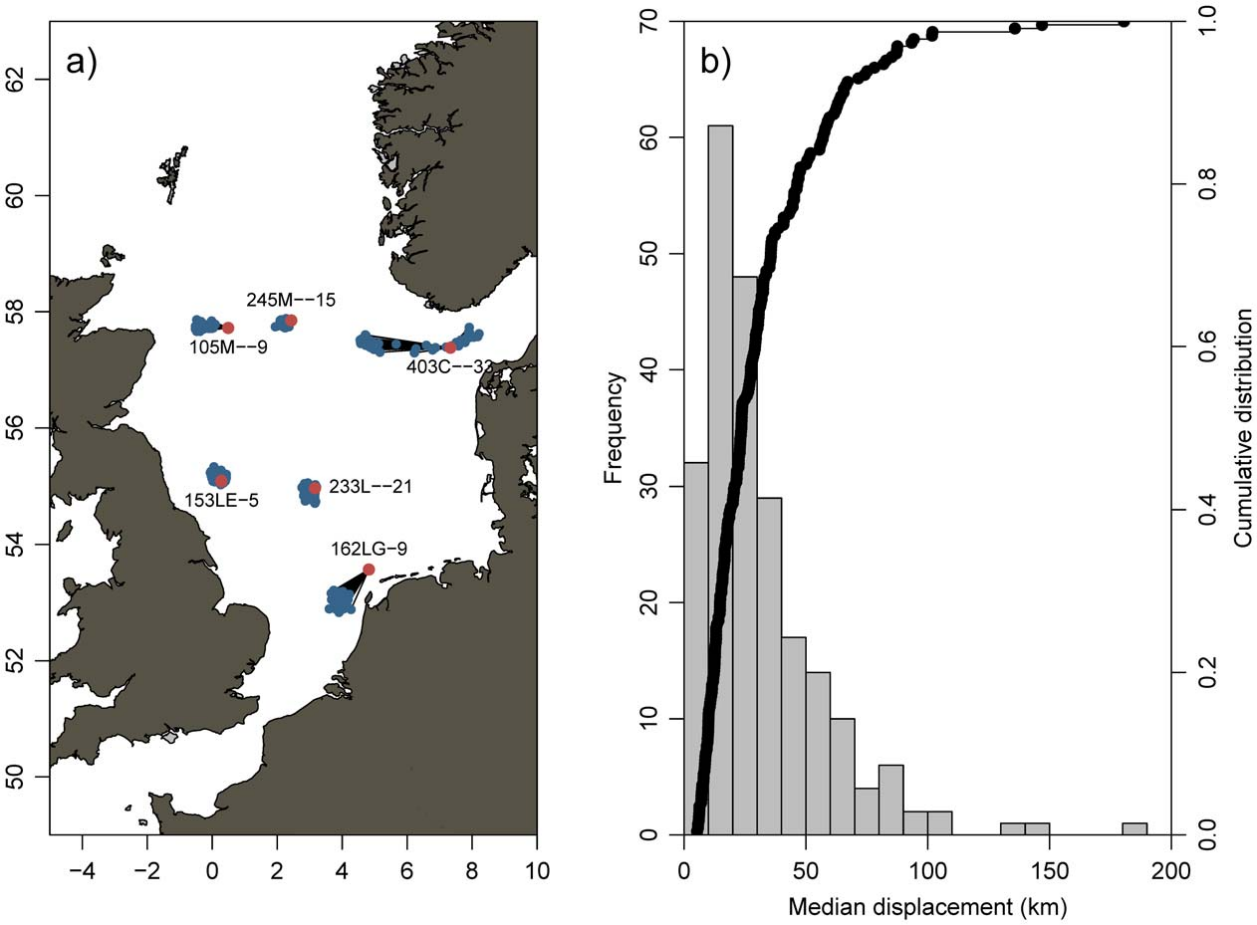
Backtracking simulations. a) Examples of backtracked trajectories for six observations of larval in the CPR distributed across the North Sea. Red circles mark capture points in the CPR, blue circles the end points of particles after 10 days of backtracking. Black lines connect the two points for visual reference. Text denotes the CPR label code. b) Distribution of particle displacements after 10 days drift. Left axis (grey bars) depict the frequency (number of CPR observations containing larvae) for each 10km class bin. Black-line with black dot (right axis) shows the empirical cumulative distribution function.

Hydrographic drift simulations showed that advection of the larvae between the estimated spawning time and capture by the CPR was generally minor (Figure 3a). 90% of the larvae caught by the CPR had drifted less than 60 km from the spawning site and 75% have drifted less than 35 km (Figure 3b). Advection of mackerel eggs and larvae between spawning and capture in the CPR, and therefore any interannual variability associated with it, can reasonably be assumed not to induce a significant bias in the spawning distribution when looking for changes at the scale of the North Sea basin. The CPR larval observations can therefore be used as proxies for the spawning distribution of North Sea mackerel.

Larvae abundance model parameters are given in Table 1. Spatial correlation was found to be 0.65 on a 100 km distance (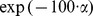). Temporal correlation between adjacent years was estimated to be 0.74 (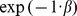). The “nugget effect” was found to be highly significant (p<0.001).

**Table 1:** Larvae model parameter estimates.

Parameter	Estimate	s.d.
_σ_^2^_0_	2.36	0.17
_σ_^2^	6.43	2.08
_Log(α)_	−5.45	0.19
_Log(β)_	−1.22	0.22
*_aspawn1_*	2.41 * 10^−1^	2.44 * 10^−2^
*_aspawn1_*	6.21 * 10^−4^	2.77 * 10^−5^
*_athcl1_*	8.33 * 10^−2^	2.46 * 10^−2^
*_athcl2_*	2.71 * 10^−3^	7.48 * 10^−4^

**Figure 4:**
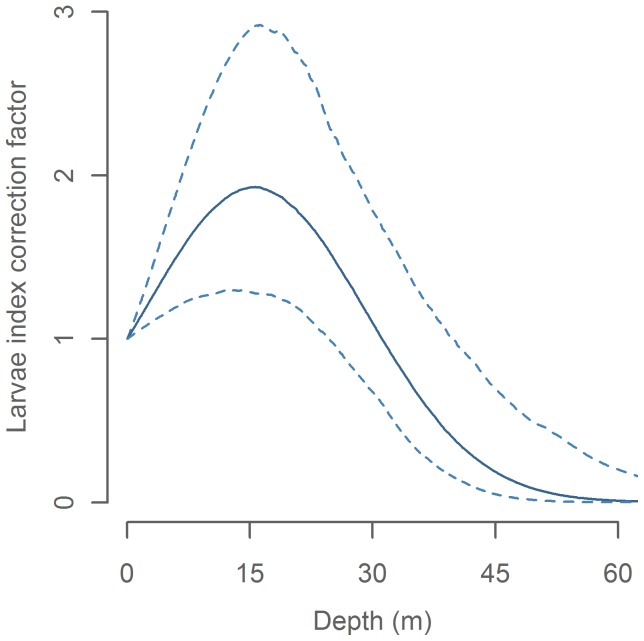
Catchability effect of thermocline depth on CPR larvae index.

Of the two catchability effects; thermocline depth was found to be significant (p<0.001) whilst the diurnal catchability pattern (hour effect) was not (p = 0.75). Consequently only thermocline depth was retained in the final model. Catchability peaked in areas where the CPR was sampling just above the thermocline. Larvae were rarely caught when the thermocline was below 45 m (Figure 4). Having corrected for catchability effects, we assume that the CPR catches represents the true larvae concentration plus random sampling error.

**Figure 5:**
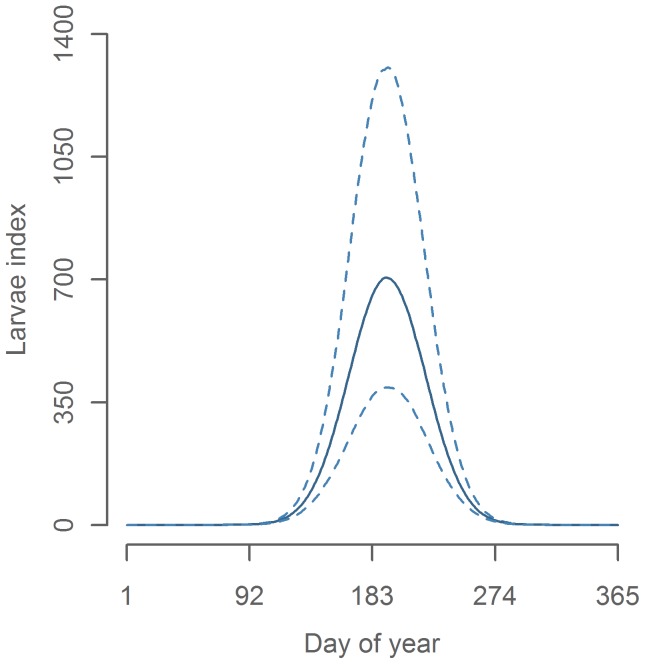
Seasonal effect on CPR larvae index.

The seasonal peak of the larvae abundance was found to be in mid-July (day number 193, see Figure 5). Since we estimated mean larval age to be approximately 10 days, this corresponds to a peak in spawning at the start of July. This is comparable to egg survey based estimates from 1982–2008, where the peak spawning were found to be 8–20 days earlier [30]. A difference in this direction were expected because our study period includes cooler decades than the period from 1982 to 2008 and spawning is known to be earlier in warm years [30].

**Figure 6:**
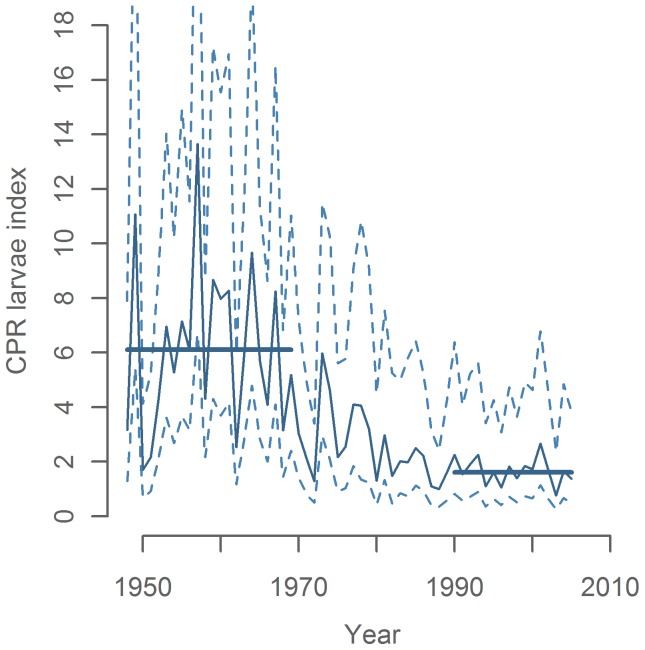
Larvae abundance index with 95% confidence interval.

**Figure 7:**
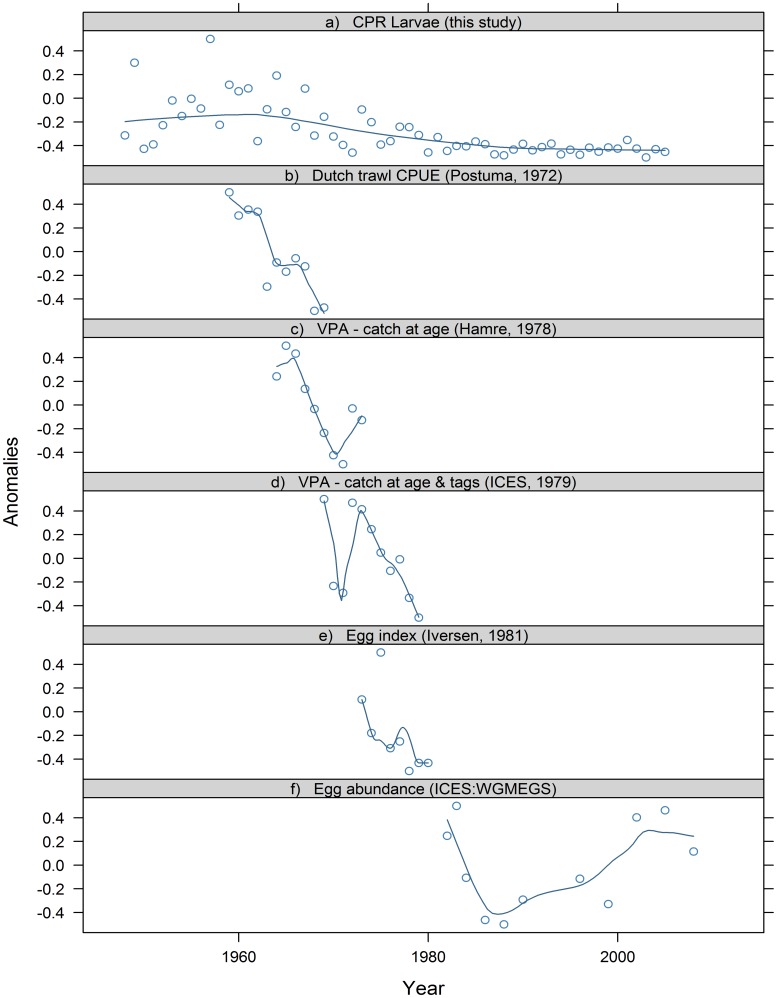
Long term mackerel trends in the North Sea [**6]**, [27]–[**29**]. Loess smoothed trend lines with span = 0.5.

Annual larvae abundance index is illustrated for the whole study period in Figure 6. We found a significant (p<0.001) shift in the mean larvae index of 6.1 from before 1970 to 1.6 after 1990 (Figure 6). There is unfortunately too much variability in the CPR larval index to precisely pinpoint the onset and completion of this decline (Figure 6; Figure 7a). Nevertheless, the broad pattern of a systematic decline in abundance between 1970 and the mid-1980s shown here agrees with data from other independent sources e.g. standardized catch rates in the Dutch commercial spring fishery and catch/tagging based assessments indicate a decline beginning in the late 1960s (Figure 7b,c). The decline continues through the 1970s, as also indicated by the catch/tagging based ICES assessment and early mackerel egg surveys (Figure 7d,e), ending the decline in the mid 1980’s. The CPR larval index is therefore in broad agreement with the piecewise picture available from other data sources: however, it also has the clear advantage of covering the entire time-span of interest.

**Figure 8:**
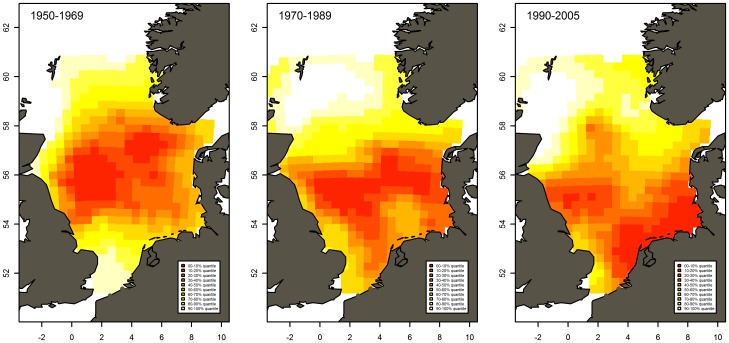
Modeled spatial distribution of mackerel larvae caught by CPR. Color scale from white (low abundance) represents values in 0–10% quantile, etc. up to red 90–100% quantile (high abundance).

Spatial distributions obtained from the model showed a shift in spawning area from early to recent decades (Figure 8 and S2), suggesting that the central North Sea is no longer as important as the areas further west and south. This change is in line with the results from the international mackerel egg surveys; although these surveys do not cover the extreme south and southeast (Figure 1) (ICES WGMEGS reports and pers. comm. S. Iversen, 13 Oct. 2011). Spawning in the north-western North Sea was, as also observed in the egg surveys, at a very low level in all periods.

## Discussion

In this work we present a unique time series describing the dynamics of the North Sea mackerel. For the first time for this stock, a single unbroken time series, based on a consistent sampling methodology with broad spatial and temporal coverage, has been presented. The time series covers the full time span of interest, from 1948, through the 1970s and 1980s stock collapse, all the way up to 2005. This index is based on a novel analysis of Continuous Plankton Recorder observations, using powerful modern statistical techniques. The resulting perspective is both unique and gives a broad view of the dynamics of this population where previously only brief glimpses were available.

Our results confirmed the long-term development of the North Sea stock, previously based on assessments of spawning stock size and egg abundance covering part of the time span. Furthermore we found a spatial shift corresponding to a similar observation in egg distribution. This provides some validation for all approaches and suggests that the larvae index, at least on longer time scales, is a usable proxy for egg abundance and spawning stock size in the North Sea.

It is noteworthy that the uncertainty and interannual variability in the CPR index was very high. Several sources of variability seem possible: i) high statistical uncertainty such as random sampling error that increase due to the few larvae being captures in the later years, ii variation in fecundity, iii) variation in mortality during the approximately 7 days of egg phase and 2 days of larval phase, iv) poor spatial sampling coverage in the central North Sea in later years, v) lack of sampling in Skagerrak/Kattegat.

However, our conclusion on the decline from before 1970 to after 1990 seems robust to these uncertainties. Even though sampling intensity in the central North Sea has been reduced in the later decades, the sampling that did take place in this area did not result in catch rates comparable to those in the earlier decades. Furthermore, analysis of the spatial patterns (Figure 8) also suggests that the central North Sea is no longer as important as the areas further west and south. However, a spatial shift back towards the central North Sea in the future might not readily be detected with the present survey design. Improved spatial coverage in this region would therefore improve the precision of the CPR larval index and further increase the value of this time series for the scientific community as well as stock advice and management.

Spawning is also known to take place in Skagerrak/Kattegat. The importance of this area is possibly limited to approximately 5% of the North Sea mackerel spawning [31]. However, this estimate is highly uncertain as the area has never been properly covered by the CPR or egg survey.

The CPR survey covered parts of the North Sea outside the egg survey area, providing an opportunity to evaluate the spatial coverage of the North Sea egg survey (Figure 1 and 8). Modelled distribution of larvae in the whole North Sea showed that the Southern North Sea has been a relatively important spawning area in the North Sea through the last decades. This result suggests that the area covered by the mackerel egg survey does not cover the entire spawning distribution, and may need to be expanded.

The described incomplete spatial coverage of both egg and larvae surveys, combined with the relatively high signal-to-noise ratio in the latter decades of low stock size, prevents us from validating the low level variation in SSB in the latter decades as suggested by the egg survey data (figure 7f).

The new time series developed herein has the potential to address several outstanding problems regarding the mackerel stock in the North Sea. The most significant of these is: “Why has the North Sea spawning stock not rebuilt despite decades of protection from commercial fisheries?”. We propose four hypothesis that may explain this observation: i) Changes in environment or predation pressure have reduced the productivity of the stock; ii) The fishing pressure is still too high due to by-catches in herring fisheries and/or in the large fishery for western mackerel in the northern North Sea; iii) The North Sea mackerel is not a separate natal homing stock and the observed collapse was merely a change in distribution of a single large north eastern Atlantic panmictic mackerel population; or iv) The North Sea mackerel was a separate natal homing stock up to the collapse where after modification of the genotype and behaviour happened as a result of intermixture between the small North Sea stock and the larger western stock [32]. Whilst it was not possible to address these questions directly here, further analysis of the CPR larval index have made a valuable contribution to testing hypothesis 3 by comparing the large interannual fluctuations with similar fluctuations in the western spawning area [33]. Furthermore, time series analysis relating the presented index with environmental factors has given indications on causal relationships between biological/physical drivers and migration [33].

Finally, phyto-, zoo- and ichtyoplankton data from the CPR survey have repeatedly been used by scientists because of the unique spatiotemporal coverage over the last 8 decades. Typical methods for compiling time series have been deterministic algorithms raising the organism count in the samples to monthly averages in designated spatial rectangles, that are then aggregated over months or rectangles to provide time series or maps [8]. Present day’s improvement in computer power has made it possible to apply advanced statistical models to large high resolution datasets, such as CPR plankton samples. Applying state-of-the-art statistical models such as the present log-gaussian cox process model provides numerous advantages over the more simple deterministic raising algorithms. Organisms as well as the CPR samples are often patchily distributed in time and space. Any analysis of CPR data should consistently deal with these challenges, estimate the uncertainty that stem from these sources and propagate it into the final result. To deal with vertical patchiness and migration, that can have great effect on the variance of the relation between densities in CPR samples at 7 m and the whole water column [9], [11], we considered two factors with potential to affect vertical distribution. By means of hypothesis testing, we could build the final model using only the significant parameter. The horizontal distribution issues were considered by using the exact sample positions (midpoints) and accounting for the spatial correlations between samples. This allowed for a more informed estimation of larvae densities in unsampled areas what could have been obtained through simple interpolations. Furthermore, it added to the uncertainty estimation procedure. Similarly, we could model temporal autocorrelation with i) a year-to-year correlation and ii) a seasonal day-to-day correlation. All model features were accounted for when maximizing likelihood of the model-observation fit. With this model we were able to provide the most likely estimate of larval density at any position and at any time – sampled or unsampled and present maps and time series in any resolution accompanied with uncertainty estimates.

We recommend the usage of such models for analyses of CPR data and encourage revisiting previously published studies with the aim of expansion and improvement.

## Supporting Information

Figure S1 **Maps of continuous plankton recorder sample locations in the spawning season May-July.** a) 1948–1959. b) 1960–1974. c) 1975–1989. d) 1990–2005.
(TIF)

Figure S2 **Animation of modeled annual spatial distribution of mackerel larvae caught by CPR.** Color scale from white (low abundance) to red (high abundance).
(SWF)
