# Supplementary figures and images for: Long-Term Retrospective Analysis of Mackerel Spawning in the North Sea: A New Time Series and Modeling Approach to CPR Data
Source: PLoS One. 2012 Jun 21;7(6):e38758. doi: 10.1371/journal.pone.0038758 (PMC3380938; doi:10.1371/journal.pone.0038758)

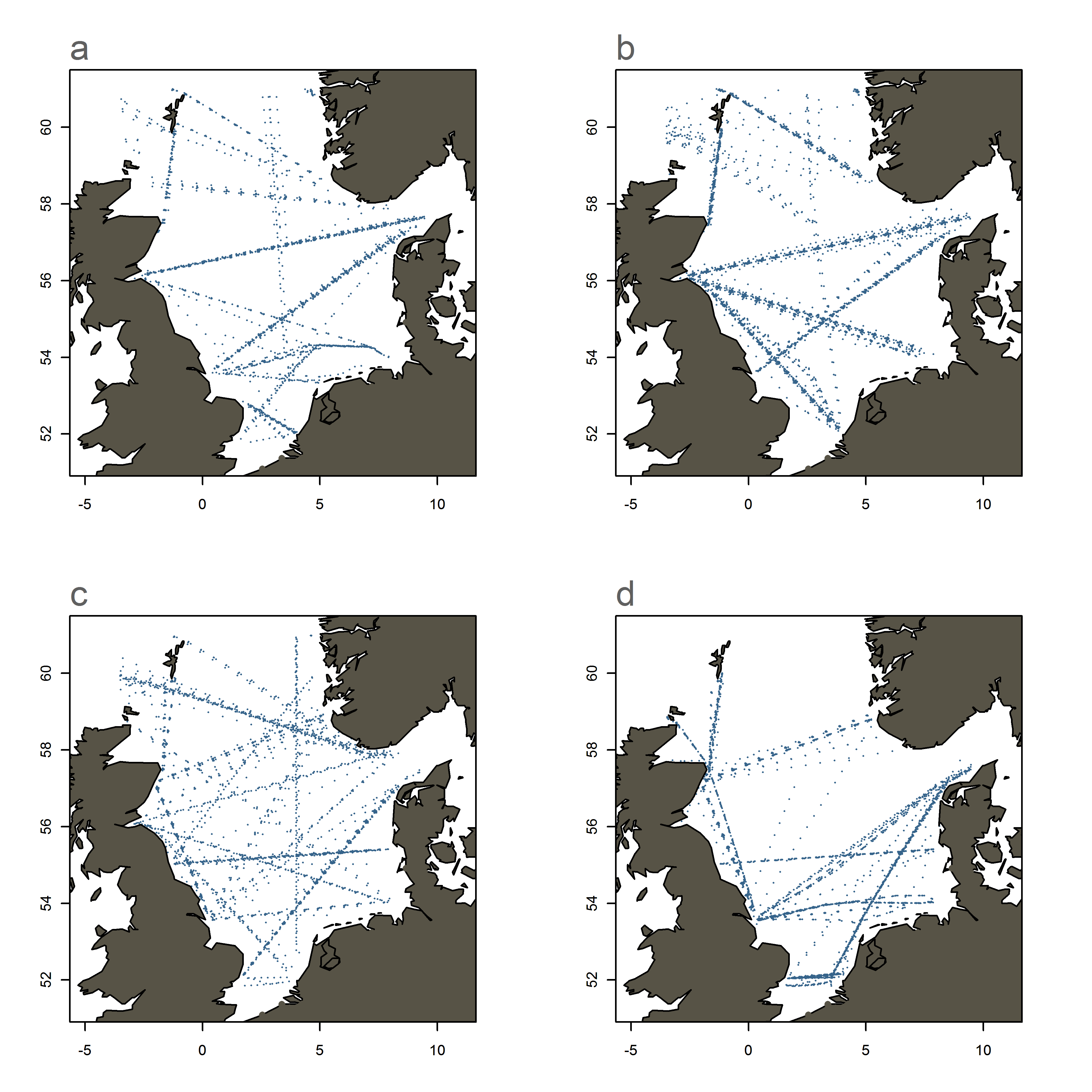

Supplement: Figure S1 — Maps of continuous plankton recorder sample locations in the spawning season May-July. a) 1948–1959. b) 1960–1974. c) 1975–1989. d) 1990–2005. (TIF) [file pone.0038758.s001.tif]
